# Allergic diseases of the skin and drug allergies – 2014. Predictive value of clinical history in suspcicion of quinolones hypersensitivity

**DOI:** 10.1186/1939-4551-6-S1-P101

**Published:** 2013-04-23

**Authors:** Luciana Kase Tanno, Anca M Chiriac, Pascal Demoly

**Affiliations:** 1 Allergy, University Hospital of Montpellier – Insern U657, Montpellier, France; 2 Allergy and Pneumology, University Hospital of Montpellier – Insern U657, Montpellier, France

## Background

Quinolones are antibiotics increasingly used over the last decade and consequently hypersensitivity reactions to these drugs are ever more described. However, not all reactions are due to the drug. Therefore, we analyze the predictive value of clinical history of quinolones hypersensitivity (QH) from our database.

## Methods

A historico-prospective cohort study was developed including all patients with history of QH consulting and tested during the last 10 years. All *in vivo* investigation followed the ENDA (*European Network of Drug Allergy*) recommendations and we considered them the reference to calculate the predictive value of history of QH.

## Results

We studied 78 patients, 55 (70%) female, the mean age was 50 years. Urticaria (26%) and maculo-papular exanthema (20%) were the most frequent manifestations; anaphylaxis was reported in 27% of cases. Ciprofloxacin (36%), Ofloxacin (20%) and Levofloxacin (18%) were the most frequent suspected drugs. Forty-one (52%) patients presented the manifestations in the first hour after the intake of the drug. The diagnosis was confirmed in 30 (39%) patients, 24 (80%) were by drug provocation test (DPT). The concordance between the symptoms referred in the clinical history and the manifestations of positive DPT was of 71%. Positivity occurred in 20 (67%) patients who experienced immediate reactions and in 10 (29%) with non-immediate reactions. The specificity of the clinical history of QH was 15% and its positive predictive value (PPV) was 42%. The PPV for those who experienced urticaria was 43% and for maculo-papular exanthema, 25%; while it was 70% for the patients who reported anaphylaxis.

## Conclusions

The accuracy of the clinical history of anaphylaxis due to quinolones showed to be higher than for other clinical patterns, but overall the PPV of the clinical history of QH demonstrated to be insufficient for the diagnosis and a drug allergy work up is needed.

